# Human Spotted Fever Group Rickettsioses Are Underappreciated in Southern Taiwan, Particularly for the Species Closely-Related to *Rickettsia felis*

**DOI:** 10.1371/journal.pone.0095810

**Published:** 2014-04-22

**Authors:** Chung-Hsu Lai, Lin-Li Chang, Jiun-Nong Lin, Kun-Hsien Tsai, Ya-Chien Hung, Li-Li Kuo, Hsi-Hsun Lin, Yen-Hsu Chen

**Affiliations:** 1 Graduate Institute of Medicine, College of Medicine, Kaohsiung Medical University, Kaohsiung City, Taiwan; 2 Division of Infectious Diseases, Department of Internal Medicine, E-Da Hospital/I-Shou University, Kaohsiung City, Taiwan; 3 Division of Infection Control Laboratory, E-Da Hospital/I-Shou University, Kaohsiung City, Taiwan; 4 Faculty of Medicine, Department of Microbiology, College of Medicine, Kaohsiung Medical University, Kaohsiung City, Taiwan; 5 Institute of Environmental Health, College of Public, Health, National Taiwan University, Taipei City, Taiwan; 6 Infectious Diseases Research and Education Center, Ministry of Health and Welfare and National Taiwan University, Taipei City, Taiwan; 7 Research and Diagnostic Center, Centers for Disease Control, Department of Health, Taipei City, Taiwan; 8 Institute of Clinical Medicine, National Yang-Ming University, Taipei City, Taiwan; 9 Division of Infectious Diseases, Department of Internal Medicine, Kaohsiung Medical University Hospital, Kaohsiung Medical University, Kaohsiung City, Taiwan; 10 School of Medicine, College of Medicine, Kaohsiung Medical University, Kaohsiung City, Taiwan; University of Texas Medical Branch, United States of America

## Abstract

**Background:**

Despite increased identification of spotted fever group rickettsioses (SFGR) in animals and arthropods, human SFGR are poorly characterized in Taiwan.

**Methods:**

Patients with suspected Q fever, scrub typhus, murine typhus, leptospirosis, and dengue fever from April 2004 to December 2009 were retrospectively investigated for SFGR antibodies (Abs). Sera were screened for *Rickettsia rickettsii* Abs by indirect immunofluorescence antibody assay (IFA), and those with positive results were further examined for Abs against *R. rickettsii*, *R. typhi*, *R. felis*, *R. conorii*, and *R. japonica* using micro-immunofluorescence (MIF) tests. Polymerase chain reaction (PCR) for detection of SFGR DNA was applied in those indicated acute infections. Case geographic distribution was made by the geographic information system software.

**Results:**

A total of 413 cases with paired serum, including 90 cases of Q fever, 47 cases of scrub typhus, 12 cases of murine typhus, 6 cases of leptospirosis, 3 cases of dengue fever, and 255 cases of unknown febrile diseases were investigated. Using IFA tests, a total of 49 cases with 47 (11.4%) and 4 (1.0%) cases had sera potentially positive for *R. rickettsii* IgG and IgM, respectively. In the 49 cases screened from IFA, MIF tests revealed that there were 5 cases of acute infections (3 possible *R. felis* and 2 undetermined SFGR) and 13 cases of past infections (3 possible *R. felis* and 10 undetermined SFGR). None of the 5 cases of acute infection had detectable SFGR DNA in the blood specimen by PCR. Possible acute infection of *R. felis* was identified in both one case of Q fever and scrub typhus. The geographic distribution of SFGR cases is similar with that of scrub typhus.

**Conclusions:**

Human SFGR exist and are neglected diseases in southern Taiwan, particularly for the species closely-related to *R. felis*.

## Introduction

Rickettsioses are a group of diseases that historically include rickettsial diseases, ehrlichiosis, anaplasmosis, scrub typhus (caused by *Orientia tsutsugamushi*), and Q fever (caused by *Coxiella burnetii*), but *Ehrlichia* and *Anaplasma* were removed from the family Rickettsiaceae, *Orientia* from the genus, and *Coxiella* from alpha-proteobacteria [1]. These diseases are common zoonoses in humans that may present as a fever of unknown origin in clinical settings. Current classification of rickettsial diseases includes 3 biogroups: spotted fever group rickettsioses (SFGR), typhus group rickettsioses (TGR), and scrub typhus [1], [2]. SFGR are found worldwide, the geographic distribution varying for different *Rickettsia* spp., and the causative rickettsiae are often named according to the location or the arthropod vectors where they are first described [1], [2]. Most SFGR are tick-borne, except *Rickettsia akari* and *R. felis*, which are mite-borne and flea-borne, respectively [1], [3].

SFGR are emerging infections, and many of the causative rickettsiae have been identified as human pathogens, but the human pathogenicity of some SFGR are unknown [1], [3], [4]. They are neglected emerging diseases, particularly in developing countries [5]–[7]. In East Asia, human SFGR infections of *R. japonica* in Japan [8]–[10], *R. conorii*, *R. japonica*, and *R. felis* in South Korea [11], [12], *R. sibirica*, *R. conorii*, *R. akari,* and *R. heilongjiangensis* in China [13], [14], and *R. sibirica* in Mongolia [15] have been identified. In Taiwan, there have been increased reports of isolation and identification of SFGR, including novel strains, from arthropods in recent years [16]–[22]. However, serological investigation of human SFGR was poorly characterized [23], [24]. Although Q fever, scrub typhus, and murine typhus are widely reported by clinicians in Taiwan [25]–[31], human SFGR infections have rarely been identified, except one imported human case of an African tick bite fever in 2009 (caused by *R. africae*) [32] and one indigenous *R. felis* infection in 2008 [33]. In addition, many suspected cases of Q fever, scrub typhus, murine typhus, leptospirosis, and dengue fever reported to Centers for Disease Control, Taiwan (Taiwan CDC) are excluded by confirmatory tests for each suspected disease. It is reasonable to speculate that SFGR might account for some of these cases that have either similar clinical manifestations or share exposure to arthropod vectors as a risk factor but are treated with agents effective against rickettsioses without appropriate diagnosis.

The aim of this study is to investigate the seroepidemiology of SFGR in patients who had suspected cases of Q fever, scrub typhus, murine typhus, leptospirosis, and dengue fever in southern Taiwan.

## Methods

### Ethics Statement

This study was approved by the Ethics Committee of the E-Da Hospital (EMRP-097-117). The committee waived the need for written informed consent because the demographic information and clinical data were retrospectively recorded, and all of the data were collected anonymously.

### Study Setting and Selection of Study Cases

**Figure 1:**
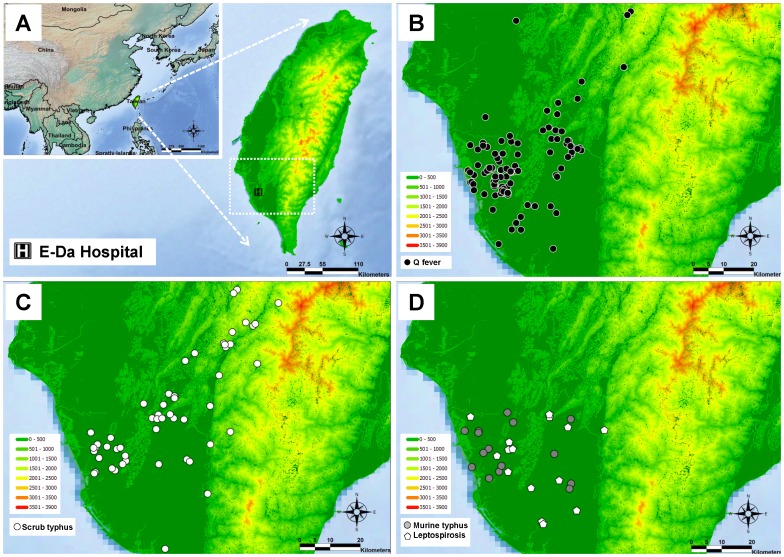
The case distribution of confirmed Q fever, scrub typhus, murine typhus, and leptospirosis. A: The geographic location of Taiwan and E-Da hospital. B: The case distribution of acute Q fever (black circles). C: The case distribution of scrub typhus (white circles). D: The case distribution of murine typhus (gray circles) and leptospirosis (white pentagons).

E-Da hospital, a regional and referral hospital comprising 1200 beds, locates at southern Taiwan (Figure 1A). Patients who were clinically suspected Q fever, scrub typhus, murine typhus, leptospirosis, and dengue fever were enrolled because these diseases are common zoonoses or arthropod-vectored diseases in Taiwan and are clinically difficult to differentiate them from SFGR. The confirmation or exclusion of each disease was determined according to the final reports of notifiable diseases of Taiwan CDC.

### Diagnosis of Q Fever, Scrub Typhus, Murine Typhus, Leptospirosis, and Dengue Fever

Q fever, scrub typhus, murine typhus, leptospirosis, and dengue fever are notifiable diseases in Taiwan, and clinicians are requested to report patients who are clinically suspected of having these diseases to Taiwan CDC. Paired blood specimens (acute and convalescent phase) are collected and sent to the contract laboratories of Taiwan CDC for confirmatory or exclusive diagnosis. Q fever was diagnosed by an anti-phase II antigen IgM titer of ≥1∶80 and a 4-fold or greater increase of anti-phase II antigen IgG titer in paired sera using indirect immunofluorescence antibody assay (IFA), or positive detection of *C. burnetii* DNA in the blood by polymerase chain reaction (PCR). Scrub typhus was diagnosed by an antibody titer of IgM ≥1∶80 and 4-fold or greater rise of total antibody (IgG+IgA+IgM) titer in paired sera for Karp, Kato, or Gilliam strain of *O. tsutsugamushi* using IFA, or positive detection of *O. tsutsugamushi* DNA in the blood by PCR. Murine typhus was diagnosed by an antibody titer of IgM ≥1∶80 and a 4-fold or greater rise of titer of IgG against to *R. typhi* in paired sera using IFA, or positive detection of *R. typhi* DNA in the blood by PCR. Dengue fever was diagnosed by a blood specimen positive for NS1 antigen, a dengue virus gene detected by PCR, or specific IgM against the dengue virus detected by enzyme-linked immunosorbent assay (ELISA). Leptospirosis was diagnosed by a 4-fold or greater increase of specific antibody in paired serum by microscopic agglutination test (MAT) or *Leptospira* spp. isolated from urine. Patients who were excluded from the initial suspected notifiable diseases by Taiwan CDC were classified as cases of unknown febrile diseases in this study.

### Screening Test for SFGR Antibodies by IFA Kits

Blood samples were comprised of the residual specimens obtained for the purpose of diagnosing Q fever, scrub typhus, murine typhus, leptospirosis, and dengue fever for the Taiwan CDC. Specimens were stored at −80°C until analysis. Serum was first screened for SFGR (*R. rickettsii*) and TGR (*R. typhi*) antibodies by a commercially available IFA kits (Rickettsia IFA IgG [IF0100G] and IgM [IF0100M], Focus Diagnostic, USA). The positive and negative controls of human serum used in the procedures were contained in the IFA kits. Assays were performed according to the manufacturer’s instructions and the test titer started at 1∶40. An antibody titer of ≥1∶80 is considered as positive reaction. Serum with an antibody titer of ≥1∶40 was considered to be potentially positive for SFGR antibodies and was included further micro-immunofluorescence (MIF) test.

### Detection of Antibodies against Selected Species of SFGR by MIF Kits

To detect antibodies to selected antigens of SGFR simultaneously, a commercially available customized MIF kits (Rickettsia screen IFA IgG [R50G-120] and IgM [R50M-120] antibody kits) (Fuller Laboratories, USA) were used. Antigens of *R. felis*, *R. japonica*, and *R. conorii* were selected because they were SFGR commonly reported in Asia and Taiwan. Antigens of *R. rickettsii* and *R. typhi* used in IFA kits were selected as well for comparison. The positive and negative controls of human serum used in the procedures were contained in the MIF kits. The procedures were performed according to the manufacturer’s instructions. The test titer started at 1∶32 and an antibody titer of ≥1∶64 is considered as positive reaction.

### Interpretation of Serological Cross-reaction and SFGR Infections

*Acute infection of SFGR*: either a 4-fold or greater increase of IgG titer in paired sera or a positive detection of IgM specific for *Rickettsia* species; *Past infection of SFGR*: either an IgG titer ≥1∶80 by IFA or ≥1∶64 by MIF without a 4-fold or greater increase of titer, and IgM negative; *Cross-reaction interpretation*: a rickettsial antigen was considered to represent the agent of infection when IgG or IgM antibody titers against this antigen were at least 2 serial dilutions higher than titers of IgG or IgM antibody against other rickettsial antigens [3], [34], [35]; *Undetermined SFGR infection*: acute or past infection of SFGR without a representative species of SFGR could be determined by the above criteria.

### Detection of SFGR DNA in Blood Specimens

The blood specimens of acute infection of SFGR were included for SFGR DNA detection. PCR tests with primers targeting 17-kDa protein gene, *groEL*, and *ompB* of *Rickettsia* spp. were performed as previously described [33]. A nested PCR was also applied to detect *gltA* and *rompB*, as described previously [11]. The DNA extracted from *R. felis* was used as the positive control for PCR assays [17].

### Clinical Characteristics and Geographic Distribution of SFGR Infection Cases

The demographic data, clinical manifestations, findings of image examinations, results of laboratory examinations, and antimicrobial treatments were recorded retrospectively by medical chart review. The map of case geographic distribution was made by marking the resident address of each case using SuperGIS Desktop software (Supergeo Technologies Inc.) and free vector and raster map data obtained from Natural Earth (a public domain map dataset, http://www.naturalearthdata.com).

## Results

### Studied Cases

From April 2004 to December 2009, a total of 413 cases clinically suspected of Q fever, scrub typhus, murine typhus, leptospirosis, and dengue fever with paired blood specimens (acute and convalescent phase) available at E-Da hospital were included in this study. According to the final reports of Taiwan CDC, 90 cases of Q fever, 47 cases of scrub typhus, 12 cases of murine typhus, 6 cases of leptospirosis, and 3 cases of dengue fever were determined. The other 255 cases were unknown febrile diseases.

### Serological Results of Antibodies against *R. rickettsii* by IFA Tests

A total of 49 (11.9%) cases, including 47 (11.4%) and 4 (1.0%) cases for IgG and IgM, respectively, had *R. rickettsii* antibody titer of ≥1∶40 in either acute or convalescent phase sera (Table 1).

**Table 1:** Titers of antibodies against *R. rickettsii* a in 413 cases of clinically suspected Q fever, scrub typhus, murine typhus, leptospirosis, and dengue fever.

Clinical final diagnosis^b^	*R. rickettsii* antibody titer, n (%)							
	> = 1∶40	1∶40	1∶80	1∶160	1∶320	1∶640	1∶1280	1∶2560
*R. rickettsii* IgG (n = 413)	47 (11.4)	28 (6.8)	12 (2.9)	2 (0.5)	2 (0.5)	3 (0.7)	0 (0)	0 (0)
Q fever (n = 90)	7 (7.8)	5 (5.6)	0 (0)	1 (1.1)	0 (0)	1 (1.1)	0 (0)	0 (0)
Scrub typhus (n = 47)	8 (17.0)	4 (8.5)	3 (6.4)	0 (0)	0 (0)	1 (2.1)	0 (0)	0 (0)
Murine typhus (n = 12)	3 (25.0)	2 (16.7)	0 (0)	0 (0)	1 (8.3)	0 (0)	0 (0)	0 (0)
Leptospirosis (n = 6)	1 (16.7)	0 (0)	0 (0)	1 (16.7)	0 (0)	0 (0)	0 (0)	0 (0)
Dengue fever (n = 3)	0 (0)	0 (0)	0 (0)	0 (0)	0 (0)	0 (0)	0 (0)	0 (0)
UFD (n = 255)	28 (11.0)	17 (6.7)	9 (3.5)	0 (0)	1 (0.4)	1 (0.4)	0 (0)	0 (0)
*R. rickettsii* IgM (n = 413)	4 (1.0)	3 (0.7)	0 (0)	1 (0.2)	0 (0)	0 (0)	0 (0)	0 (0)
Q fever (n = 90)	0 (0)	0 (0)	0 (0)	0 (0)	0 (0)	0 (0)	0 (0)	0 (0)
Scrub typhus (n = 47)	0 (0)	0 (0)	0 (0)	0 (0)	0 (0)	0 (0)	0 (0)	0 (0)
Murine typhus (n = 12)	3 (25)	3 (25.0)	0 (0)	0 (0)	0 (0)	0 (0)	0 (0)	0 (0)
Leptospirosis (n = 6)	0 (0)	0 (0)	0 (0)	0 (0)	0 (0)	0 (0)	0 (0)	0 (0)
Dengue fever (n = 3)	0 (0)	0 (0)	0 (0)	0 (0)	0 (0)	0 (0)	0 (0)	0 (0)
UFD (n = 255)	1 (0.4)	0 (0)	0 (0)	1 (0.4)	0 (0)	0 (0)	0 (0)	0 (0)

a Indirect immunofluorescence antibody assay (Rickettsia IFA IgG and IgM, Focus Diagnostic, USA).

b According to the final reports of Taiwan CDC.

UFD = Unknown febrile disease.

### Serological Results of Antibodies against Selected Species of SFGR and Detection of SFGR DNA

Of the 49 cases with *R. rickettsii* antibody titer of ≥1∶40 by IFA tests, MIF tests revealed that there were 5 cases of acute infection of SFGR (3 *R. felis* and 2 undetermined SFGR) and 13 cases of past infection of SFGR (3 *R. felis* and 10 undetermined SFGR) (Table 2). There were 9 cases of cross-reaction caused by infection of *R. typhi* (the titer of *R. typhi* was at least 4-fold greater than other tested *Rickettsia* spp.), and 22 cases were excluded as not infected by the tested *Rickettsia* spp. (titer was 1∶40 by IFA, but non-reactive by MIF tests). Among the 5 cases of acute infections, all had acute phase sera and only one (case 1) had acute phase peripheral blood monocytes (PBMCs) available for PCR test. However, none had detectable SFGR DNA.

**Table 2:** Results^a^ of micro-immunofluorescence (MIF) tests^b^ of patients who are positive for *R. rickettsii* IgG or IgM by indirect immunofluorescence antibody assay (IFA) tests^c^.

		IFA IgG IFA IgM		MIF IgG					MIF IgM				
		SFGR		SFGR				TGR	SFGR				TGR
Clinical final diagnosis^d^		*R. rickettsia*		*R. rickettsii*	*R. conorii*	*R. japonica*	*R. felis*	*R. typhi*	*R. rickettsii*	*R. conorii*	*R. japonica*	*R. felis*	*R. typhi*
Acute infection of SFGR													
Case 1	UFD	0/80	0/0	0/32	0/128	0/128	0/256	0/0	0/0	0/0	0/0	0/0	0/0
Case 2	UFD	40/320	0/160	0/1024	0/1024	0/2048	**0/4096** *	0/0	0/128	0/32	0/0	**0/256** *	0/0
Case 3	Q fever	0/160	0/0	0/64	0/64	0/64	**0/256** *	0/0	0/0	0/0	0/0	0/0	0/0
Case 4	Scrub typhus	80/640	0/0	128/512	512/512	256/256	**1024/4096** *	0/0	0/0	0/0	0/0	0/0	0/0
Case 5	UFD	0/640	0/0	0/512	0/2048	0/2048	0/4096	0/0	0/0	0/0	0/0	0/0	0/0
Past infection of SFGR													
Case 6	UFD	80/80	0/0	0/0	32/32	32/32	32/32	0/32	0/0	0/0	0/0	0/0	0/0
Case 7	UFD	0/80	0/0	32/0	0/0	0/0	0/32	0/0	0/0	0/0	0/0	0/0	0/0
Case 8	UFD	0/80	0/0	0/0	0/0	0/0	0/32	0/0	0/0	0/0	0/0	0/0	0/0
Case 9	UFD	40/80	0/0	32/32	32/32	32/64	**128/64** *	32/64	0/0	0/0	0/0	0/0	0/0
Case 10	Q fever	160/640	0/0	32/32	0/32	0/32	**128/128** *	0/0	0/0	0/0	0/0	0/0	0/0
Case 11	Scrub typhus	0/40	0/0	0/32	0/64	64/64	**128/256** *	0/0	0/0	0/0	0/0	0/0	0/0
Case 12	Q fever	0/40	0/0	32/64	0/0	0/0	32/64	0/0	0/0	0/0	0/0	0/0	0/0
Case 13	Q fever	40/40	0/0	32/32	64/64	64/64	128/128	32/32	0/0	0/0	0/0	0/0	0/0
Case 14	Leptospirosis	0/160	0/0	0/0	0/0	0/0	0/0	0/0	0/0	0/0	0/0	0/0	0/0
Case 15	UFD	40/80	0/0	0/0	0/0	0/0	0/0	0/0	0/0	0/0	0/0	0/0	0/0
Case 16	UFD	80/0	0/0	0/0	0/0	0/0	0/0	0/0	0/0	0/0	0/0	0/0	0/0
Case 17	UFD	80/80	0/0	0/0	0/0	0/0	0/0	0/0	0/0	0/0	0/0	0/0	0/0
Case 18	Scrub typhus	40/80	0/0	0/0	0/0	0/0	0/0	0/0	0/0	0/0	0/0	0/0	0/0

a Presentation of antibody titers: acute phase titer/convalescent phase titer.

b Customized micro-immunofluorescence assay (Rickettsia screen IFA IgG and IgM antibody kit, Fuller Laboratories, USA).

c Indirect immunofluorescence antibody assay (Rickettsia IFA IgG and IgM, Focus Diagnostic, USA).

*Considered as the representative pathogen of infection.

SFGR = Spotted fever group rickettsiae; TGR = Typhus group rickettsiae; UFD = Unknown febrile disease.

### Clinical Characteristics of Acute Infection of SFGR

The clinical characteristics of the 5 cases of acute infection of SFGR and one previously reported case are summarized in Table 3. The most common manifestations were fever, chills, headache, elevated liver enzymes, and thrombocytopenia. Only one (case 2) had itching skin rash during the course of presentation. However, it was suspected to be an allergic reaction to moxifloxacin by the clinical assessment. Two had history of animal contact, but none had reported contact with cats or bitten by insects or arthropods. Case 3 and case 4 were Q fever and scrub typhus, respectively, confirmed by Taiwan CDC.

**Table 3:** Clinical characteristics of cases of acute infection of spotted fever group rickettsioses (SFGR) in Taiwan.

Case No.	Case 1	Case 2	Case 3	Case 4	Case 5	Other case [33] a
Clinical finaldiagnosis	UFD	UFD	Q fever	Scrub typhus	UFD	NA
Identified SFGR	UndeterminedSFGR	Possible *R. felis*	Possible *R. felis*	Possible *R. felis*	UndeterminedSFGR	*R. felis*
Age/Sex	48/F	50/M	31/M	67/M	40/M	27/F
Underlying diseases	Nil	Alcoholism	Alcoholism, HCV	HBV, HCV, cirrhosis,diabetes mellitus	Nil	Nil
Clinical symptomsand signs	Fever, chills,	Fever, chills,headache, skinrash, relativebradycardia	Fever, chills,headache, abdominalpain, myalgia,relative bradycardia	Fever, chills, cough,abdominal pain,myalgia, relativebradycardia	Fever, chills,headache	Fever, chills,headache, fatigue,acute polyneuropathy
Resident in mountainarea/rural	Nil	Yes	Yes	Yes	Nil	Nil
Recent travel in mountain/rural area	Yes	Nil	Nil	Nil	Nil	Nil
Animal contact history	Dogs, chicken	Nil	Cattle	Nil	Nil	Nil
Insects/arthropods bites	Nil	Nil	Nil	Nil	Nil	Nil
Chest x-ray	Normal	Normal	Normal	Increased infiltrationover right lower lung	Normal	Normal
Abdominal ultrasonography	Fatty liver	Fatty liver	Fatty liver,hepatosplenomegaly	Cirrhosis, splenomegaly,fatty liver, gallstonesand polyps	Fatty liver	NA
Laboratory examination	Normal bloodcell count,elevatedliver enzymes(ALT/AST:122/188 U/L)	Thrombocytopenia(platelet count:113000/uL),elevated liverenzymes (ALT/AST:155/191 U/L)	Leukocytosis(WBC:14210/uL),elevated liverenzymes(ALT/AST:150/214 U/L)	Thrombocytopenia(platelet count:149000/uL), elevatedliver enzymes (ALT/AST:108/113 U/L)	Thrombocytopenia(69000/uL), elevatedliver enzymes(ALT/AST:208/46 U/L)	Normal blood cellcount, liver enzymes
Antimicrobial treatment	Doxycycline	Moxifloxacin,doxycycline	Doxycycline	Doxycycline,levofloxacin	Doxycycline	Doxycycline

a Case 6 was reported by Tsai *et al*. [33].

Normal range of blood examinations: WBC (3600∼10600/uL), platelet count (150000∼400000 uL), ALT (0∼40 U/L), AST (0∼38 U/L).

UFD = Unknown febrile disease; HBV = Hepatitis B virus; HCV = Hepatitis C virus; ALT = Alanine transaminase; AST = Aspartate transaminase; WBC = White blood cell count; NA = Not available.

### The Geographic Distribution of Q Fever, Scrub Typhus, Murine Typhus, Leptospirosis, and SFGR

**Figure 2:**
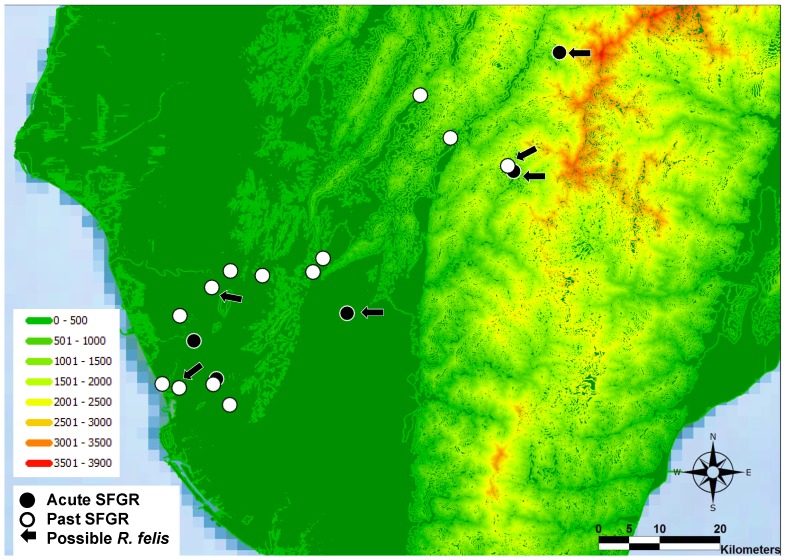
The case distribution of spotted fever group rickettsioses (SFGR) infections. Black circles: The 5 cases of acute infection SFGR. White circles: The 10 cases of past infection of SFGR. Black arrows: The 6 cases of possible *Rickettsia felis* infection.

The geographic distribution of cases of Q fever, scrub typhus, murine typhus, and leptospirosis are illustrated in Figure 1 (B∼D). Except nearly half of scrub typhus cases were distributed in mountainous areas, most cases of Q fever, murine typhus, and leptospirosis were distributed in plains areas. Figure 2 illustrates the distribution of the 18 cases of SFGR infections listed in Table 2. The cases of SFGR infections were distributed in both mountainous and plains areas, similar to scrub typhus.

## Discussion

SFGR is poorly characterized in Taiwan, particularly regarding human infections. Only two human serologic studies of SFGR conducted approximately 2 decades ago [23], [24]. During 1983 to 1989, 3.5 to 4.4% of sera samples from 113 residents in Tainan City, located in southern Taiwan, were positive for the *R. sibirica* antibody [23]. In 1995, none of 548 cases suspected as scrub typhus and murine typhus had serum that was positive for *R. rickettsii* and *R. conorii* antibodies [24]. Recent studies identified several species of SFGR closely related to *R. rhipicephali* [16], [21], *R. australis* [16], *R. japonica* [20], *R. conorii* [20], and *R. felis* [16]–[18], [20], [21], or novel strain of SFGR [22] in arthropods trapped from cats, dogs, or rodents. In additional, serological study in small animals had revealed infections of *R. felis* in cats [17] and *R. conorii* in rodents [19]. However, despite increased identification of SFGR in animals and arthropods, no further indigenous human SFGR was reported after the first case of human *R. felis* infection identified in 2008 [33]. The results of this retrospective serological investigation illustrate that human SFGR infections do exist in Taiwan, but may be underappreciated.

*Rickettsia felis*, a rickettsiae belonging to SFGR, is the causative pathogen of so-called flea-borne spotted fever (or cat-flea typhus), which is regarded as an emerging global rickettsiosis [34], [36]–[38]. Although isolation or molecular evidence of *R. felis* has been reported in fleas, ticks, mites, and mosquito [39], the cat flea (*Ctenocephalides felis*) is the only known biological vector of *R. felis* [40]. However, direct evidence of transmission from *C. felis* to animals has not been determined and no viable *R. felis* has been isolated from a vertebrate host yet [36], [40]. The clinical manifestation of human infection with *R. felis* is similar to murine typhus (caused by *R. typhi*) or other rickettsioses, which makes the clinical diagnosis difficult [38]. In Asia, emerging human *R. felis* infections have been reported in the Thai-Myanmar border [35], South Korea [11], Laos [41], and Taiwan [33]. In our study, most cases of SFGR infections have higher antibody titers against *R. felis* than other tested *Rickettsia* spp., including 3 acute and 3 past infections of *R. felis* by serological interpretation (Table 2). This is consistent with the recent findings derived from investigations of *R. felis* in animals and arthropods in Taiwan [16]–[18], [20], [21]. However, no SFGR DNA was detected in this study. This might due to only one case had acute phase PBMC obtained after administration of doxycycline and the others had sera available for PCR examination. In a critical view, however, we just have identified human infection of SFGR close-related to *R. felis* in Taiwan for lack of direct molecular evidence or isolation of *R. felis*.

Serological diagnosis of SFGR is challenge because cross-reaction is notoriously found between the species of SFGR. Western blot assays and cross-absorption studies are the recommended methods to distinguish species, but they demand large amounts of antigens derived from different *Rickettsia* specie [1]. Even in national reference centers, most antigens of SFGR may not be available for testing and Taiwan as well [3]. Comparing with IFA, MIF can simultaneously detect antibodies to a number of rickettsial antigens with the same drop of serum in a single well containing multiple rickettsial antigen dots [42]. For consideration of cost and availability of reagents, we used commercial available IFA and MIF kits for serological screening and identification of potentially representative SFGR, respectively. However, regarding to *R. rickettsii* antibodies, some cases of SFGR infections might be missed if either IFA or MIF were used only (Table 2). Although difference in titers between antigens was applied for interpreting serological cross-reaction as previous literatures [3], [34], [35], the higher titers of *R. felis* and non-reaction by MIF in our cases could be related to other species close-related to *R. felis* (such as *R. australis* and *R. akari*) and those species not used in this study or still unknown in Taiwan, respectively. This indicates the complexity of serological cross-reaction between species of SFGR, and variant sensitivity and specificity of serological reagents for different SFGR species.

The most common manifestations of the 5 cases of acute SFGR infections and one other previously reported case were fever, chills, headache, elevated liver enzymes, and thrombocytopenia (Table 3), which are similar to the symptoms of other rickettsioses in Taiwan [28], [29], [31]. Among the 5 cases, 3 were possible *R. felis* (case 2, 3, and 4) and the other 2 (case 1 and 5) were undetermined SFGR infections. All of them had received antimicrobial agents effective against rickettsioses and none had been suspected SFGR and reported to Taiwan CDC for confirmatory test. According, cases of human SFGR might be well treated in clinical without appropriate and definitive diagnosis in Taiwan.

Notably, 2 of the 5 cases of acute SFGR infections were also confirmed with Q fever (case 3) and scrub typhus (case 4) by Taiwan CDC. Among the 13 case of past SFGR infections, 3 cases were Q fever, 2 were scrub typhus, and one was leptospirosis (Table 3). Although Q fever had been reported to serologically cross-reactive with *R. conorii* and *R. typhi* [43], most of the our cases had higher antibody titer of *R. felis* than *R. conorii,* and were non-reactive to *R. typhi* (Table 3). Concomitant infection with Q fever and tick-borne rickettsioses has been reported, including *R. conorii* [44], [45], *R. slovaca* [44], and *R. africae* [44]. In southern Taiwan, concomitant infections with Q fever and scrub typhus [46], murine typhus and Q fever, and murine and scrub typhus [31] were identified. This indicates that patients suspected or confirmed with zoonosis or arthropod-vectored diseases might also have been exposed to the environment, animal, or arthropod vectors containing various SFGR [16]–[21], and are at risk of infection by SFGR in southern Taiwan. The clinicians have neglected this possibility and not conducted diagnostic approaches for SFGR. The overlapping distribution of zoonosis and SFGR cases illustrated by Figures 1 and 2, particularly of scrub typhus, could partially support this speculation. The present study not only proves human SFGR existing in Taiwan, but also provides the first evidence of possible concomitant infection of SFGR (possible *R. felis*) with Q fever and scrub typhus in Taiwan.

This retrospective serology and clinical study has limitations. Only antigens of 5 *Rickettsia* spp. were tested, and other potential SFGR might be unrecognized. Due to the lack of isolation and antigens of tested *Rickettsia* spp., Western blot assays and cross absorption tests were not available for further identification of the responsible SFGR. Because this was a retrospective study on residual blood specimens, not all acute infection cases had appropriate specimens for the molecular detection of SFGR, and the antibody reactivity might have decayed with time from collection to test.

This work illuminates that human SFGR infection does exist and is unappreciated in southern Taiwan where Q fever, scrub typhus, murine typhus, leptospirosis, and dengue fever are endemic. This observation, accompanied by increasing SFGR detected in animals and arthropods, indicates that human SFGR infection should not be overlooked, particularly for *R. felis*. Announcement and education regarding SFGRs, especially for clinicians and populations at high risk of acquisition, could be helpful for uncovering potential human cases. Meanwhile, establishment of standardized diagnostic methods and the inclusion of SFGR as notifiable diseases would be helpful for better characterizing these emerging diseases in Taiwan.
